# Optimizing Aesthetic Results in Implant-Based Breast Reconstruction: Patient Factors, Surgical Techniques, and Adjunctive Strategies

**DOI:** 10.3390/jcm14197106

**Published:** 2025-10-09

**Authors:** Elisa Bertulla, Edoardo Raposio

**Affiliations:** 1 Department of Surgical Sciences and Integrated Diagnostics (DISC), University of Genova, L.go R. Benzi 10, 16132 Genoa, Italy; edoardo.raposio@unige.it; 2 Plastic and Reconstructive Surgery Division, IRCCS Ospedale Policlinico San Martino, 16132 Genoa, Italy

**Keywords:** implant-based breast reconstruction, breast reconstruction, aesthetic outcome

## Abstract

**Background**: Breast reconstruction following mastectomy plays a crucial role in breast cancer management, restoring physical form and significantly impacting psychological well-being and quality of life. Implant-based breast reconstruction (IBBR) is the most performed technique worldwide due to its relative simplicity, shorter operative times, and avoidance of donor site morbidity. Achieving satisfactory aesthetic outcomes, however, remains challenging, as multiple factors—including patient characteristics, surgical technique, implant selection, timing of reconstruction, and adjuvant therapies—can influence the final appearance. **Methods**: Literature research was performed via PubMed, Scopus and Cochrane Library Database, focusing on studies examining aesthetic outcomes in implant-based breast reconstruction published between 2015 and 2025. Data on type of study, sample size, aesthetic evaluation methods, and duration of follow-up were collected and summarized. **Results**: Among 747 articles identified, only 25 articles fulfilled inclusion criteria, including mostly retrospective studies, but also prospective studies, randomized clinical trials, and reviews. Factors such as BMI, inframammary fold management, and implant selection were consistently reported to influence aesthetic outcomes. Surgical techniques including ADM use, axillary advancement sutures, hybrid reconstruction with fat grafting, and prepectoral implant placement were associated with improved patient satisfaction. Patient satisfaction often differs from surgeon-assessed outcomes, emphasizing the importance of subjective evaluation. **Conclusions**: Despite the heterogeneity and retrospective nature of many studies, evidence indicates that optimizing aesthetic outcomes in IBBR relies on careful patient selection, tailored surgical planning, and meticulous use of evidence-based techniques, including implant selection, flap-based support, and adjunctive strategies. Patient-reported outcomes are essential for evaluating success, and future research should focus on standardized outcome measures and prospective studies to further refine reconstructive approaches and maximize both cosmetic satisfaction and quality of life.

## 1. Introduction

Breast reconstruction following mastectomy plays a crucial role in the holistic management of breast cancer patients, not only restoring physical form but also significantly impacting psychological well-being and quality of life [1].

Among the available reconstructive options, implant-based reconstruction remains the most commonly performed technique worldwide due to its relative simplicity, shorter operative times, and avoidance of donor site morbidity [2]. This approach can be performed either in a retropectoral plane, beneath the pectoralis major muscle, or in a prepectoral plane, above the muscle, each with specific implications for aesthetic outcomes and complication profiles [3]. The use of mesh has since become a common component of most immediate implant-based breast reconstructions (IBBR) to reinforce the soft tissue, offering a scaffold for revascularization and ensuring additional coverage and support for the implant [4,5]. Reconstruction can be performed simultaneously with mastectomy as a one- or two-stage procedure, or it can be delayed and performed as a two-stage procedure [6].

However, achieving satisfactory aesthetic outcomes with IBBR remains a complex challenge, as multiple factors—including patient characteristics, surgical techniques, implant selection, and adjuvant therapies—can influence the final appearance of the reconstructed breast. Despite its widespread use, aesthetic outcomes can vary considerably depending on patient-specific factors such as breast size, skin and soft tissue thickness, body mass index, and previous or planned adjuvant therapies, particularly radiotherapy [7]. Additional determinants include surgical technique, implant selection, and the handling of mastectomy flaps, all of which contribute to breast contour, symmetry, and projection [8]. It is, therefore, essential to have careful patient selection and preoperative planning, considering factors such as skin quality, chest wall anatomy, and patient expectations.

Furthermore, various strategies have been proposed to enhance aesthetic outcomes, including meticulous surgical techniques, careful implant selection, and the use of adjunctive materials like acellular dermal matrices and fat grafting [9].

Understanding these factors and strategies is essential to improve breast contour, symmetry, and patient satisfaction, which are increasingly recognized as critical endpoints in modern reconstructive practice.

This review aims to provide an overview of the factors influencing aesthetic outcomes in IBBR and to discuss strategies for optimizing these results, thereby enhancing patient satisfaction and quality of life.

## 2. Materials and Methods

Literature research was performed via PubMed, Medline, and Cochrane Library Databases according to Preferred Reporting Items for Systematic Reviews and Meta-Analysis (PRISMA) guidelines [10], with the aim of providing a comprehensive review of the literature about studies examining aesthetic outcomes in implant-based breast reconstructions. Although this work is not a systematic review, we have chosen to follow PRISMA guidelines to enhance the rigor and transparency of our methodology and to increase the validity of our findings. To obtain more current evidence, only studies published in the past 10 years (2015–2025) were included.

The following MeSH terms were used: (“implant-based breast reconstruction”) AND (“aesthetic guidelines” OR “cosmetic guidelines” OR “aesthetic criteria” OR “aesthetic recommendations” OR “cosmetic recommendations”) between 5 September 2025 and 8 September 2025.

Titles and abstracts were screened to identify eligible articles.

The selection of the studies was based on the following inclusion criteria:

- Studies focusing on implant-based breast reconstruction;
- Studies addressing aesthetic standards or techniques to improve cosmetic outcomes related to implant-based reconstruction;
- Original research articles, systematic and narrative reviews, clinical guidelines;
- Articles published between 2015 and 2025;
- Full texts available in English.

The studies were excluded due to any one of the following criteria:

- Studies focusing on autologous or combined reconstruction;
- Studies focusing exclusively on cosmetic procedures or breast surgery for congenital malformations;
- Studies discussing only surgical techniques without reference to aesthetic outcomes;
- Articles addressing breast aesthetics unrelated to implant-based reconstruction;
- Case reports, editorials, commentaries, letters to the editor, surveys;
- Articles published before 2015;
- Articles not available in English.

Data extraction included study design, sample size, methods of aesthetic outcome assessment, principal findings related to aesthetic guidelines, and the techniques or interventions employed to optimize cosmetic results in implant-based breast reconstruction (IBBR).

## 3. Results and Discussion

**Figure 1:**
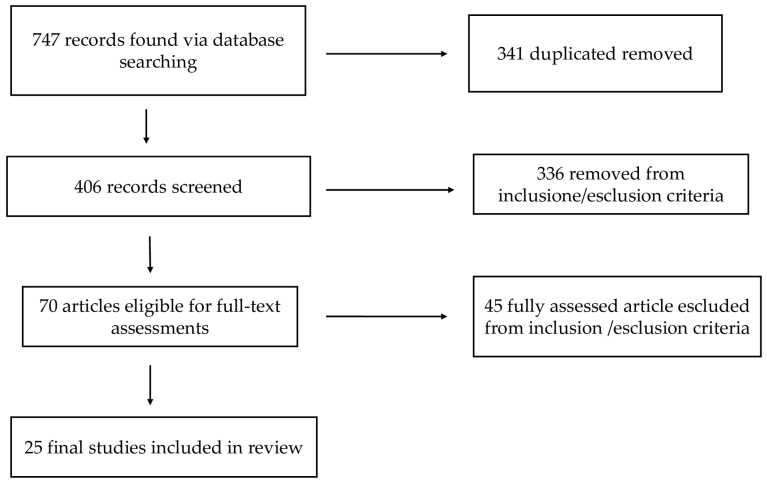
PRISMA guidelines flowchart.

A total of 747 citations were initially identified from PubMed, Scopus, and the Cochrane Library. Of these, 341 duplicates were removed. Articles published before 2015 and articles not available in English were also excluded. After screening titles and abstracts, 70 records were considered potentially relevant. Full-text review further excluded 45 articles. Ultimately, 25 articles published between 2015 and 2025 met the inclusion criteria and were included in the review (Figure 1, PRISMA Guidelines).

The analyzed literature mainly consisted of retrospective clinical studies, three prospective studies, two randomized controlled trials (RCTs) and seven reviews. The studies included variable numbers of patients, ranging from small cohorts of 50 patients to larger series of 1000 cases, reflecting the diversity of study designs and surgical techniques employed.

All the analyzed articles are listed in Table 1.

In our study, we observed several factors influencing aesthetic outcomes in implant-based breast reconstruction. In our society, breasts are considered essential components of female beauty and sexuality. Any change to a woman’s natural form can have a negative impact on her self-esteem and body image [34]. Therefore, achieving satisfactory cosmetic results is crucial not both psychological and physical recovery.

### 3.1. Factors Influencing Aesthetic Outcomes in Implant-Based Reconstruction

#### 3.1.1. Body Mass Index (BMI) and Nipple Areola Complex (NAC) Preservation

Although the effect varies based on patient characteristics, BMI seems to affect aesthetic outcomes in direct-to-implant reconstruction. Two studies analyzed the influence of BMI on aesthetic outcomes in DTI, including a total of 976 patients. In the first study by Ukleikins et al. [12], a moderate negative correlation was demonstrated between BMI and aesthetic outcome, as well as BMI and volume differences between breasts.

Highlighting that cases with increased BMI are particularly challenging to achieve good aesthetic satisfaction and volume symmetry with implant-based IBR only and emphasizing the need for autologous immediate breast reconstruction availability in the unit for optimal patient satisfaction.

In the second study by Vaccari et al. [11], in contrast with previous studies, a correlation was demonstrated between underweight status and the development of capsular contracture, which could compromise aesthetic outcome. Conversely, no significant difference was observed between overweight/obese patients and the normal-weight group. This study also revealed that underweight patients experienced significantly greater rates of rippling and implant visibility. These findings may be explained by the influence of soft-tissue thickness and quality: lower BMI or thinner tissue coverage increases the risk of visible implant edges and rippling. Adjunctive measures, such as fat grafting or ADM use, could be considered to optimize soft tissue coverage.

In the current literature, individuals with low BMI are often grouped together with those of normal weight, which limits understanding of the specific risks faced by this population [11].

All studies agree that, as expected, nipple preservation considerably contributes to improved aesthetic outcomes [12]. However, in skin-reducing mastectomy (SRM), its feasibility and impact are less well established due to the extensive skin excision, which may compromise vascularization. This aspect was not specifically addressed in the studies reviewed and warrants investigation in future research.

When nipple preservation is not feasible, NAC reconstruction has been shown to enhance patient satisfaction following implant-based breast reconstruction, as measured by the BREAST-Q questionnaire [35].

Overall, these findings underscore the importance of tailoring reconstructive planning to individual BMI and tissue characteristics.

#### 3.1.2. Mesh Use vs. Non-Use and Synthetic vs. Biological

Two randomized controlled trials (RCTs) by Lohmander et al. [13] and Negenborn et al. [14], including a total of 256 patients demonstrated no greater aesthetic satisfaction with the use of acellular dermal matrix (ADM) when comparing IBBR with and without ADM (both DTI and two-stage), although slight cosmetic differences in favor of ADM emerged in two subscale items, as well as when comparing one-stage versus two-stage ADM-based reconstruction.

A 2018 review by DeLong et al. [4] focused exclusively on aesthetic outcomes of submuscular expander-to-implant breast reconstructions with ADM compared to muscular coverage. In the context of subpectoral breast reconstruction, comparing ADM to muscular coverage effectively represents a comparison of ADM use vs. non-use, since total muscular coverage is the standard approach in the absence of ADM, which is used to complete the muscle pocket when the inferior border of the pectoralis muscle is unable to cover the lower pole of the implant. When assessments were based on patient satisfaction, no differences were reported between ADM and muscular reconstruction. In contrast, studies using objective measures demonstrated a significant improvement in aesthetic outcomes in the ADM group.

A retrospective study by Blohmer et al. [16] reported high aesthetic satisfaction among both surgeons and patients in reconstructions using ADM, although no comparison group was included, highlighting the generally positive patient perception associated with ADM use.

According to Nahabedian et al. [36], the application of ADM enhances the definition of the inframammary fold, contributing to improved aesthetic outcomes in IBBR. Makarewicz et al. [15] in a review from 2023 evaluating biological versus synthetic meshes in IBBR concluded that synthetic meshes were rated as at least equivalent to their biological counterparts across all reported outcomes. These findings are confirmed by a review by Amro et al. [31].

Mesh use in implant-based reconstruction offers soft tissue support, a scaffold for revascularization, and enhanced implant coverage [37]. While randomized trials and reviews show no consistent improvement in patient-reported aesthetic satisfaction with ADM compared to muscular coverage, objective evaluations suggest a potential benefit in cosmetic outcomes. ADM may also enhance the definition of the inframammary fold, contributing to improved aesthetics. Synthetic meshes seem to perform at least as well as biological meshes across reported outcomes, indicating that both options are feasible based on surgical preference and patient factors. Further high-quality comparative studies, including long-term follow-up, are warranted to clarify the optimal mesh type and reconstructive approach for achieving consistent cosmetic results.

#### 3.1.3. Type of Implant

In a 2019 study by Agochukwu-Nwubah et al. [17] 156 patients undergoing IBBR were divided into two groups based on implant shape: 123 received round implants and 33 received anatomical implants. No differences were observed between the groups in terms of satisfaction and well-being, as assessed by the BREAST-Q survey. However, other studies have reported that anatomic implants are associated with a lower incidence of capsular contracture, a factor that significantly impacts aesthetic outcomes [17].

A study by Vorstenbosch [18] involving 1077 patients indicated that postoperative breast satisfaction and health-related quality of life after immediate implant-based breast reconstruction appear to be unaffected by implant surface type. Nonetheless, smooth implants were associated with a higher likelihood of rippling. On the other hand, a review by Amro et al. [31] reports a study showing that micro-textured implants improve outcomes by reducing the rate of capsular contracture.

While overall patient satisfaction appears similar between round and anatomical implants, anatomical implants may reduce the incidence of capsular contracture, potentially improving long-term aesthetic outcomes. Similarly, implant surface type does not seem to substantially affect patient-reported satisfaction or quality of life, although smooth implants may tend to exhibit visible rippling. These observations imply that implant selection should be customized, taking into consideration patient anatomy, risk factors, and surgeon preference, while maintaining the potential for favorable aesthetic results.

Regarding ideal breast size, Raposio et al. [38,39] reported that, in their European study population, patients tend to prefer larger breast sizes, suggesting that plastic surgeons should consider patients’ perceptions and preferences to manage expectations and optimize satisfactory reconstruction outcomes, while acknowledging that preferences may vary across different cultural and individual contexts.

#### 3.1.4. Timing of Reconstruction

According to the 2017 retrospective study by Gschwantler-Kaulich [20] that included 170 patients, immediate implant-based breast reconstruction with direct-to-implant procedures showed a trend toward superior cosmetic outcomes, although it was associated with a higher complication rate. Kuroda et al. [21] confirmed this trend with their cross-sectional study, highlighting that age over 70 and radiotherapy are considered indicators of poorer cosmetic outcomes after immediate reconstruction.

Immediate reconstruction may provide slightly better cosmetic outcomes compared to delayed approaches, although it can be associated with a higher complication rate. When thinking about IBBR, patient selection and meticulous perioperative planning are crucial, as older age and PMRT seem to be risk factors for less-than-ideal aesthetic outcomes.

#### 3.1.5. The Inframammary Fold (IMF)

The inframammary fold (IMF) is a key determinant of aesthetic outcomes in reconstructed breasts. Tomita et al. [19] included 75 patients, who underwent unilateral two-stage reconstruction, in a retrospective study to identify factors that could be significant predictors of better IMF outcomes such as contralateral breast ptosis, absence of postmastectomy radiotherapy (PMRT), less invasive breast surgeries such as nipple-sparing mastectomy (NSM) and skin-sparing mastectomy (SSM), and larger implant volume (limited to patients without PMRT). In contrast, the timing of reconstruction did not significantly influence IMF outcomes, indicating that planning and IMF management are more important for aesthetic results. These findings support the use of strategies aimed at optimizing IMF definition, such as ADM placement or dermal sling techniques, to enhance breast shape and symmetry.

### 3.2. Strategies to Improve Aesthetic Outcomes in Implant-Based Reconstruction

#### 3.2.1. Axillary Advancement Suture

Patients undergoing IBBR following skin-sparing mastectomy (SSM) frequently report bulging in the upper flank or inferior axillary region. A preventive maneuver has been described by Lee et al. [32], that can be performed at the time of immediate reconstruction, uses an advancement suture to mobilize adjacent fat from the lateral flank pocket to cover the lateral pectoral area and the superolateral portion of the implant. This approach was shown to improve aesthetic outcomes compared to a control group. Addressing contour irregularities at the time of reconstruction can help to reduce the need for secondary procedures such as liposuction or fat grafting, although its effectiveness may be limited in patients with very low body fat or after radical procedures where insufficient tissue is available.

#### 3.2.2. Endoscopic Reverse-Order Mastectomy with a Single Axillary Incision

Yang et al. [33] proposed the use of an endoscopic reverse-order mastectomy with a single axillary incision, followed by subpectoral implant-based reconstruction. In this approach, the steps of subpectoral reconstruction are performed in a reverse dissection order, from deep to superficial layers (“subpectoral–retro-mammary space–subcutaneous”), thereby optimizing preservation of the skin envelope and nipple–areolar complex and minimizing visible scarring.

In an evaluation of 68 patients, excellent aesthetic satisfaction was reported, particularly due to the low-visibility scar—especially in patients with smaller breasts, where scars would otherwise be more conspicuous.

#### 3.2.3. Techniques in Skin-Reducing Mastectomy (SRM)

In a study by Ellabban et al. [25] involving 42 patients, the mobilization of the medial and lateral ends of the dermal flap and wrapping it as a hammock around the implant was performed to improve breast contour and compensate for discrepancies between the spared skin and the reconstructed breast mound in case of SRM.

Within the context of SRM, a 2018 review by Jepsen et al. [26] evaluated patient-reported aesthetic satisfaction in wise-pattern skin-reducing mastectomies using a dermal sling combined with an implant. This helps maintain the inframammary fold and provides an additional layer of vascularized tissue for protection. The review reported high levels of patient satisfaction and found no significant difference in aesthetic outcomes between the use of a dermal sling and ADM.

Together, these techniques highlight the importance of intraoperative maneuvers in significantly affecting long-term cosmetic satisfaction in implant-based reconstruction, emphasizing the value of customized surgical planning, careful flap management, and the implementation of innovative strategies to maximize aesthetic results, reduce the need for secondary interventions, and enhance long-term patient satisfaction.

#### 3.2.4. Nipple–Areolar Complex (NAC) Malposition

Following nipple-sparing mastectomy with implant-based reconstruction, malposition is still a common aesthetic consequence. It typically manifests as cranial displacement [40]. In a study including 88 patients by Mendoza et al. [22], the following factors were associated with vertical NAC displacement: subpectoral implant placement, use of acellular dermal matrix (ADM), immediate reconstruction, and breast-splitting mastectomy incisions. A study based on mathematical morphological simulation by Shiraishi et al. [23] demonstrated that insufficient expansion of the upper pole is a key cause of NAC malposition, particularly in large and ptotic breasts. The authors, therefore, suggested using round expanders instead of anatomical ones, fixing the NAC in a more inferior position, and possibly pulling the nipple inferiorly.

Mastectomy volume was also identified as a risk factor in a separate study by Komiya et al. [24], which additionally highlighted the following contributors: periareolar mastectomy incision with lateral extension, vertical radical mastectomy incisions, older age, and a history of radiotherapy. This study also proposed a technique that appears effective in reducing the incidence of this aesthetic complication: fixing the NAC to the pectoralis major fascia at the time of tissue expander insertion, establishing the proper NAC position preoperatively.

When discussing reconstructive options with patients, surgeons should take these risk factors into account because preventative actions can lower the need for follow-up corrective procedures and increase patient satisfaction over the long run.

#### 3.2.5. Mastectomy Scar Location

Incision scar location was evaluated as a factor influencing cosmetic appearance. In the study by Suzuki et al. [27], patients were divided into two groups: those with scars along the margins of the breast (MB group) and those with scars within the breast area (IB group). Cosmetic outcomes were assessed using a Japanese version of the SCAR-Q questionnaire (patient-reported method) and Manchester Scar Scale (MSS, objective evaluation tool). While results from the MSS shows no difference between the two groups, SCAR-Q showed that patients in the MB group reported significantly higher satisfaction compared to the IB group. However, surgeon-reported outcomes did not show any significant differences between the groups, highlighting a potential discrepancy between professional evaluation and patient perception. These findings underscore the importance of considering, if possible, scar placement during surgical planning, using the SCAR-Q, as even subtle differences in incision location can meaningfully impact patient satisfaction. They also stress how important it is to use patient-reported outcomes alongside surgeon assessments when evaluating aesthetic results in implant-based reconstruction.

#### 3.2.6. The Use of Fat Grafting

The use of fat grafting combined with implant-based breast reconstruction (hybrid reconstruction) was evaluated in a 2025 review by Muntean et al. [28] including 17 studies with a total of 730 patients. Overall aesthetic satisfaction was high. Compared to control groups (IBBR only or fat grafting only), the hybrid reconstruction group achieved higher aesthetic scores for overall appearance, volume, and contour, although no significant improvement was noted for projection. No significant differences were observed between patient-reported and surgeon-reported satisfaction regarding aesthetic outcomes. Fat grafting can enhance skin quality and help create a better soft tissue envelope, which is key for achieving the ideal breast shape, especially in irradiated patients, and may allow for the use of smaller implants.

Despite these advantages, potential risks—including oncogenic concerns, possible interference with tumor surveillance, and difficulties in distinguishing post-grafting calcifications from neoplastic calcifications on imaging—underscore the need for careful patient selection and long-term monitoring [28]. Overall, hybrid reconstruction represents a valuable strategy to enhance aesthetic outcomes while minimizing implant size, but its implementation should be carefully balanced with oncologic considerations and appropriate imaging follow-up.

#### 3.2.7. Position of the Implant (Subpectoral vs. Prepectoral)

Implant placement is a key factor influencing both cosmetic outcomes and complication rates in breast reconstruction. The traditional retropectoral approach, where the implant is placed beneath the pectoralis major muscle, frequently results in pain, muscle-related discomfort, and animation deformity. The prepectoral technique was developed to address these problems by positioning the implant just beneath the skin and subcutaneous tissue, above the muscle. By restoring the implant to the breast’s original anatomical position, this method can provide results that appear more natural [3,8].

A 2023 review by King et al. [3] reported that the incidence of animation deformity—a complication negatively affecting breast aesthetics and quality of life—was significantly higher in the retropectoral group compared to the prepectoral group. This phenomenon results from the dynamic interaction between the implant and the overlying muscle; the implant can appear to move upward and toward the axilla when patients flex their pectoral muscle and often causes breast asymmetry [41]. Revision surgery to the prepectoral plane with ADM coverage is an effective treatment for animation deformity.

Previous studies have highlighted patient interest in alternative initial procedures that could avoid animation deformity entirely [3], although some preventive strategies have been proposed in the broader literature—such as prepectoral implant placement with ADM coverage, pectoralis muscle splitting or sectioning techniques, chemical or mechanical muscle denervation [42,43]—high-quality studies specifically assessing their impact on aesthetic outcomes are lacking.

On the other hand, a common aesthetic complication of prepectoral reconstruction is contour irregularities, such as rippling (although larger series with longer follow-up are needed to definitively compare its incidence with subpectoral placement). Fat grafting has been shown to effectively correct step-offs associated with prepectoral implants, creating a more natural breast slope. Additionally, the use of ADM may reduce the incidence of this complication, as it provides increased support and stability to the implant, particularly at the inferior pole, helping to prevent bottoming out.

All studies agree that aesthetic complications can be minimized if patient selection is performed according to recommended criteria, through careful preoperative and intraoperative assessment.

The following recommendations, all based on Srinivasa et al. [33], recommend waiting at least six months after the completion of radiotherapy, as it has been shown that radiation can impair wound healing for a prolonged period. Fat grafting has been proposed to improve tissue quality and minimize radiation-induced damage. For patients requiring post-mastectomy radiotherapy (PMRT), immediate reconstruction with a tissue expander is recommended, which can then be replaced with a permanent implant prior to the start of radiotherapy if needed. Previous studies suggest that overall aesthetic outcomes may be suboptimal when radiation is delivered to an expander; however, long-term rates of capsular contracture are significantly lower with expander radiation compared to radiation to a permanent implant. Some authors have also suggested that prepectoral implant placement significantly reduces the risk of capsular contracture in the context of PMRT. Prepectoral reconstruction allows for more predictable aesthetic results following radiation, as the dimensions of the soft tissue envelope are preserved in the surgically established position despite radiotherapy.

To prevent implant migration in prepectoral reconstruction, it is recommended that during expander filling, the expander be underfilled by approximately 100–200 cc relative to the anticipated final implant size. This approach allows the final implant to be placed into a tighter pocket, reducing implant mobility and minimizing visible rippling caused by excess or redundant upper pole skin. Additionally, implant selection plays a crucial role. The use of silicone implants, rather than saline implants, reduces the risk of visible rippling. It is also advisable to use implants fully filled and cohesive gel implants, both of which are more resistant to rippling.

Finally, Lee et al. [30] developed a technique that achieves full implant coverage by using two smaller, appropriately positioned (crossed) ADMs instead of a single larger one. No significant differences in outcomes were observed, making this a viable alternative to the conventional method. According to Lee et al. [30], the implant width should initially be selected to be 1–2 cm smaller than the measured breast width, with appropriate height and projection. Once the implant has been chosen, ADMs should be selected so that 2x. Since the use of ADM alone may increase the risk of seroma formation, it is important to create slit incisions in both vertical and horizontal directions to facilitate proper fluid drainage and tissue integration.

Overall, these findings underscore that prepectoral reconstruction, when combined with careful patient selection, optimized surgical technique, and appropriate use of adjunctive strategies such as ADM and fat grafting, can provide superior aesthetic outcomes while minimizing common complications associated with implant-based reconstruction.

Based on the available evidence, the best candidates for prepectoral reconstruction are patients with small breasts or macromastia seeking a reduction in size, low-grade ptosis, smaller implant volumes, those requiring post-mastectomy radiotherapy (PMRT), and individuals wishing to reduce the risk of animation deformity or capsular contracture. Retropectoral reconstruction remains advisable in patients with high-grade ptosis or larger breasts that are not intended to be reduced, in patients requiring additional tissue support, as well as those who want to reduce their risk of rippling or needing further fat grafting procedures. Comorbidities such as diabetes, obesity, and active smoking raise the risk of complications, emphasizing the importance of careful preoperative assessment and optimization. Additional difficulties arise from radiation therapy, which affects cosmetic results and delays wound healing. While immediate reconstruction with a tissue expander can be used before PMRT, prepectoral reconstruction may allow for more predictable long-term outcomes, as the soft tissue envelope retains its surgically established dimensions even after radiotherapy.

Careful patient selection, appropriate implant choice, and consideration of adjuvant treatments such as radiotherapy are crucial to optimizing results.

When preventive strategies such as ADM coverage, pectoralis muscle management, or staged reconstruction are feasible, they should be integrated into surgical planning, although the current studies assessing these interventions are mostly retrospective, involve small sample sizes, have limited follow-up, and lack standardized outcome measures. Therefore, although these techniques appear promising, high-quality comparative studies specifically evaluating their impact on aesthetic outcomes are still lacking. Surgeons should discuss potential benefits and limitations with patients to manage expectations and guide individualized reconstructive strategies.

#### 3.2.8. Implant Infections

It is important to note that complications such as infection or threatened implant exposure can profoundly compromise long-term aesthetic results. Implant infection has severe consequences as the loss of prosthesis, and the ensuing inflammation may result in loss of the cavity and scarring. Various implant salvage tools have been described, including aggressive antibiotic therapy, surgical washout and debridement, use of negative wound pressure therapy [44], use of ADM or capsular/tissutal flaps to reinforce soft-tissue coverage and staged procedures [45,46] aimed at preserving the implant whenever feasible. Applying these measures can help preserve the implant and maintain optimal long-term aesthetic outcomes.

All these strategies may not be universally applicable, particularly in low-resource settings, where alternative surgical approaches or cost-effective modifications may be necessary.

In interpreting the aesthetic outcomes reported across studies, it is important to consider the methods used for cosmetic evaluation. The BREAST-Q was the only patient-reported outcome measure in nearly all of the studies that were part of this review. Since aesthetic evaluation is subjective by nature and dependent on a variety of patient and surgical factors, there is currently no widely recognized gold standard, despite the existence of both subjective and objective assessment tools.

This is particularly relevant in breast reconstruction, where patient satisfaction—not only oncologic safety—is a major determinant of quality of life [21]. Interestingly, research has revealed that patient-reported satisfaction often differs from surgeons or software-based evaluations, which typically do not detect significant differences; in most cases, patients express greater levels of satisfaction than those deduced from objective evaluations [47]. When assessing reconstructive success, these factors emphasize the necessity of integrating standardized, validated patient-reported measures and interpreting cosmetic results considering patient perceptions.

Despite these findings, heterogeneity in study design, patient populations, and outcome measures—particularly the reliance on subjective assessments such as BREAST-Q—limits the ability to draw definitive conclusions. Moreover, long-term follow-up and larger prospective studies are needed to better quantify the impact of specific techniques and materials on both patient- and surgeon-reported aesthetic outcomes.

Tailoring surgical planning to each patient’s anatomy, risk factors, and goals, together with the thoughtful use of adjunctive techniques, seems key to achieving the best cosmetic results in implant-based reconstruction. Future research should aim to standardize outcome reporting and explore new strategies to further improve cosmetic satisfaction while minimizing complications.

The main limitations of this review are the retrospective nature of most of the included studies and restricted database search, as the analysis was conducted using only three major databases (although they are the most used). This narrow search may have led to the omission of relevant studies and introduced potential selection bias. In addition, the quality of each included study was not formally assessed, which may further limit the generalizability of our findings to all settings. Future reviews, including additional databases and grey literature, such as conference proceedings, academic dissertations, and government reports, may provide a more comprehensive picture.

## 4. Conclusions

Aesthetic outcomes in implant-based breast reconstruction are influenced by multiple patient- and surgery-related factors, including BMI, breast size, implant type and position, nipple–areolar complex preservation, and adjunctive techniques such as ADM, dermal slings, and fat grafting. Cosmetic outcomes and patient satisfaction can be maximized through careful patient selection, customized surgical planning, and appropriate application of these techniques. Future studies with standardized outcome measures and long-term follow-up are needed to refine reconstructive strategies and further improve aesthetic outcomes.

## Figures and Tables

**Table 1:** Studies included in the review.

References	Design	N. of Patients	N. of Breasts	N. of Studies *	Outcome Measure	Follow Up **
Clinical Studies						
Vaccari et al., 2023 [11]	Retrospective analysis	921	1046	NA	Surgeon-reported (photographic assessments)	12 months
Ukleikins et al., 2019 [12]	Retrospective analysis	55	58	NA	Questionnaire	47 months (min. 8)
Lohmander et al., 2020 [13]	Randomised controlled trial	135	NR	NA	BREAST-Q and BRR26	24 months
Negenborn et al., 2018 [14]	Randomised controlled trial	121	NR	NA	BREAST-Q and surgeon-reported (photographic assessments)	12 months
DeLong et al., 2019 [4]	Sistematic review	504	NR	9	NA	NA
Makarewicz et al., 2023 [15]	Sistematic review	NR	NR	12	NA	NA
Blohmer et al., 2021 [16]	Prospective analysis	84	112	NA	Surgeon-reported (photographic assessments)	12 months
Agochukwu-Nwubah et al., 2019 [17]	Retrospective analysis	156	NR	NA	BREAST-Q	24 months
Vorstenbosch et al., 2021 [18]	Retrospective analysis	1077	NR	NA	BREAST-Q	24 months
Tomita et al., 2016 [19]	Retrospective analysis	75	75	NA	Surgeon-reported (photographic assessments)	6 months after implant exchange
Gschwantler-Kaulich et al., 2018 [20]	Retrospective analysis	180	NR	NA	Surgeon-reported (photographic assessments) and BREAST-Q	46 months (mean)
Kuroda et al., 2016 [21]	Cross-sectional study	94	NR	NA	Surgeon-reported (photographic assessments), BREAST-Q and BCCT	36.2 months
Mendoza et al., 2024 [22]	Obvservational sutdy	88	NR	NA	NR	NR
Shiraishi et al., 2024 [23]	Retrospective analysis	89	NR	NA	Surgeon-reported (photographic assessments	Minimum 12 months
Komiya et al., 2021 [24]	Retrospective analysis	78	NR	NA	Surgeon-reported (photographic assessments	6 months
Ellabban et al., 2020 [25]	Prospective analysis	42	52	NA	NR	12 months (mean)
Jepsen et al., 2019 [26]	Sistematic review	879	1184	24	NA	NA
Suzuki et al., 2024 [27]	Prospective study	57	NR	NA	SCAR-Q_J, Manchester scar scale, EORTC-QLQ	NR
Muntean et al., 2025 [28]	Sistematic review	730	NR	17	NA	20.23 (mean)
King et al., 2023 [3]	Review article	NR	NR	NR	NA	NR
Srinivasa et al., 2019 [29]	Review article	NR	NR	NR	NA	NR
Lee et al., 2019 [30]	Retrospective study	23	NR	NA	KNUH-Q	12 months
Amro et al., 2024 [31]	Review article	NR	NR	NR	NA	NA
Expert recommendations/innovation techniques						
Lee et al., 2017 [32]	Innovation technique	53	NR	NA	NA	NA
Yang et al., 2023 [33]	Innovative technique	68	NR	NA	Harris scale, Surgeon-reported (photographic assessments)	32 months

* The column ‘N. of studies’ is reported only for reviews. NA = Not Applicable. NR = Not reported. ** Follow-up: duration of follow-up in months. ‘Mean’ indicates the average follow-up, “min” the minimum follow-up reported. BREAST-Q = BREAST-Q Patient-Reported Outcome Measure; BCCT = BCCT.core software; EORTC-QLQ = European Organisation for Research and Treatment of Cancer Quality of Life Questionnaire; SCAR-Q = SCAR-Q Patient-Reported Outcome Measure; KNUH-Q = KNUH Kyungpook National University Hospital Breast Reconstruction Satisfaction Questionnaire.

## Data Availability

All data come from previously published studies, which are cited in the article.
